# Chloroquine and Hydroxychloroquine Use During Pregnancy and the Risk of Adverse Pregnancy Outcomes Using Real-World Evidence

**DOI:** 10.3389/fphar.2021.722511

**Published:** 2021-08-02

**Authors:** Anick Bérard, Odile Sheehy, Jin-Ping Zhao, Evelyne Vinet, Caroline Quach, Sasha Bernatsky

**Affiliations:** ^1^ Research Center, CHU Sainte-Justine, Montreal, QC, Canada; ^2^ Faculty of Pharmacy, University of Montreal, Montreal, QC, Canada; ^3^ Faculté de médecine, Université Claude Bernard Lyon 1, Lyon, France; ^4^ Faculty of Medicine, McGill University, Montreal, QC, Canada; ^5^ Faculty of Medicine, University of Montreal, Montreal, QC, Canada

**Keywords:** chloroquine, hydroxychloroquine, pregnancy, COVID-19 pandemic, adverse pregnancy outcomes, Quebec pregnancy cohort

## Abstract

**Introduction:** Chloroquine (CQ) and hydroxychloroquine (HCQ) are currently used for the prevention/treatment of malaria, and treatment of systemic lupus erythematosus (SLE), and rheumatoid arthritis (RA). Although present data do not show their efficacy to treat COVID-19, they have been used as potential treatments for COVID-19. Given that pregnant women are excluded from randomized controlled trials, and present evidence are inconsistent and inconclusive, we aimed to investigate the safety of CQ or HCQ use in a large pregnancy cohort using real-world evidence.

**Methods:** Using Quebec Pregnancy Cohort, we identified women who delivered a singleton liveborn, 1998–2015, (*n* = 233,748). The exposure time window for analyses on prematurity and low birth weight (LBW) was the second/third trimesters; was any time during pregnancy; only first trimester exposure was considered for analyses on major congenital malformations (MCM). The risk of prematurity, LBW, and MCM (overall and organ-specific) were quantified using generalized estimation equations.

**Results:** We identified 288 pregnancies (0.12%) exposed to CQ (183, 63.5%) or HCQ (105, 36.5%) that resulted in liveborn singletons; CQ/HCQ was used for RA (17.4%), SLE (16.3%) or malaria (0.7%). CQ/HCQ was used for 71.8 days on average [standard-deviation (SD) 70.5], at a dose of 204.3 mg/d (SD, 155.6). We did not observe any increased risk related to CQ/HCQ exposure for prematurity (adjusted odds ratio [aOR] 1.39, 95%CI 0.84–2.30), LBW (aOR 1.11, 95%CI 0.59–2.06), or MCM (aOR 1.01, 95%CI 0.67–1.52).

**Conclusion:** in this large CQ/HCQ exposed pregnancy cohort, we saw no clear increased risk of prematurity, LBW, or MCM, although number of exposed cases remained low.

## Introduction

Chloroquine phosphate (CQ) or hydroxychloroquine sulfate (HCQ) are anti-malarial drugs used in the prevention and treatment of malaria in endemic regions. They are also used to treat systemic lupus erythematosus (SLE) and rheumatoid arthritis (RA). CQ/HCQ have been shown to have antiviral effects against several viruses (Tsai et al., 1990; Pardridge et al., 1998; Savarino et al., 2001). CQ and HCQ are affordable drugs, with known efficacy and safety profiles for the authorized indications (Mejia Torres et al., 2013; Schrezenmeier and Dörner, 2020), and are on the list of essential medications published by the World Health organization (WHO) (World Health Organization, 2019). The 2020 American College of Rheumatology Guideline for the Management of Reproductive Health in Rheumatic and Musculoskeletal Diseases recommends the continued use of CQ/HCQ during pregnancy for women with SLE and RA (Sammaritano et al., 2020). Because of their anti-viral and anti-inflammatory properties, they have been proposed as potential therapy for COVID-19 infections. Although data do not show their efficacy to treat COVID-19, they have been used as potential treatments (Chen et al., 2020; Cortegiani et al., 2020; Emergency Use Authorizati, 2020; Gautret et al., 2020).

Although CQ/HCQ have known safety profiles, its elimination half-life in pregnancy is 11 days, and CQ/HCQ can be detected in plasma for more than 42 days (Chen et al., 2006; Moore and Davis, 2018). CQ/HCQ crosses the placental barrier, raising concerns of ototoxic and retinotoxic defects, mostly based on animal studies (Costedoat-Chalumeau et al., 2003) but may actually reduce the risk of congenital heart block in high-risk populations (Motta et al., 2005). However, a meta-analysis in SLE patients showed no retinopathy concerns in human pregnancies (Gaffar et al., 2019). In contrast to previous studies (Buchanan et al., 1996; Costedoat-Chalumeau et al., 2003; Motta et al., 2005; Ingster-Moati and Albuisson, 2010; Diav-Citrin et al., 2013; Kaplan et al., 2016; Kroese et al., 2017; Bermas et al., 2018), two recent studies on CQ/HCQ use during pregnancy are large population-based and methodologically sound but the results are inconclusive regarding the association between HCQ exposure and the risk of major congenital malformation (MCM) of the infants (Andersson et al., 2020; Huybrechts et al., 2021). In addition, although there are previous studies on the risk of gestational CQ/HCQ use during pregnancy, they are usually restricted to sub-populations with SLE or RA, and do not consider CQ/HCQ use as a whole adjusting for indication bias. Therefore, we aimed to assess the effect of CQ/HCQ exposure during pregnancy on the occurrence of prematurity, LBW, and MCM using real-world data.

## Materials and Methods

### Study Cohort

The study was conducted within the Quebec Pregnancy Cohort (QPC), which is a population-based cohort with prospective data collection on all pregnancies covered by the Quebec Prescription Drug Insurance, from January 01, 1998 to December 31, 2015 (presently, the QPC includes data from 1998 to 2015, and will be updated soon) (Bérard and Sheehy, 2014). Individual-level information for all pregnant women and children are obtained from province-wide databases and linked using unique personal identifiers (Supplementary Figure S1). The first day of the last menstrual period (LMP) is defined using data on gestational age, which is validated with ultrasound measures in patients’ charts (Vilain et al., 2008). Prospective follow-up is available from 1 year before LMP, during pregnancy, and until December 31, 2015; children are followed from birth until December 31, 2015 (Supplementary Figure S2).

The QPC data sources include the medical claims database (RAMQ: diagnoses, procedures, indicators of socio-economic status), Quebec Prescription Drug Insurance database (drug name, start date, dosage, duration), hospitalization archive database (MedEcho: diagnoses/procedures), and Quebec Statistics database (ISQ: patient socio-demographics, gestational age, and birth weight). Birth weight in ISQ has been found to be valid (Vilain et al., 2008). The RAMQ medication database in the QPC represents 36% of women between 15–45 years of age (Régie de l'Assurance Mala, 2015). Validation studies have shown that publicly insured pregnant women have similar characteristics and co-morbidities with those who have private medication insurance (Bérard and Lacasse, 2009).

**FIGURE 1:**
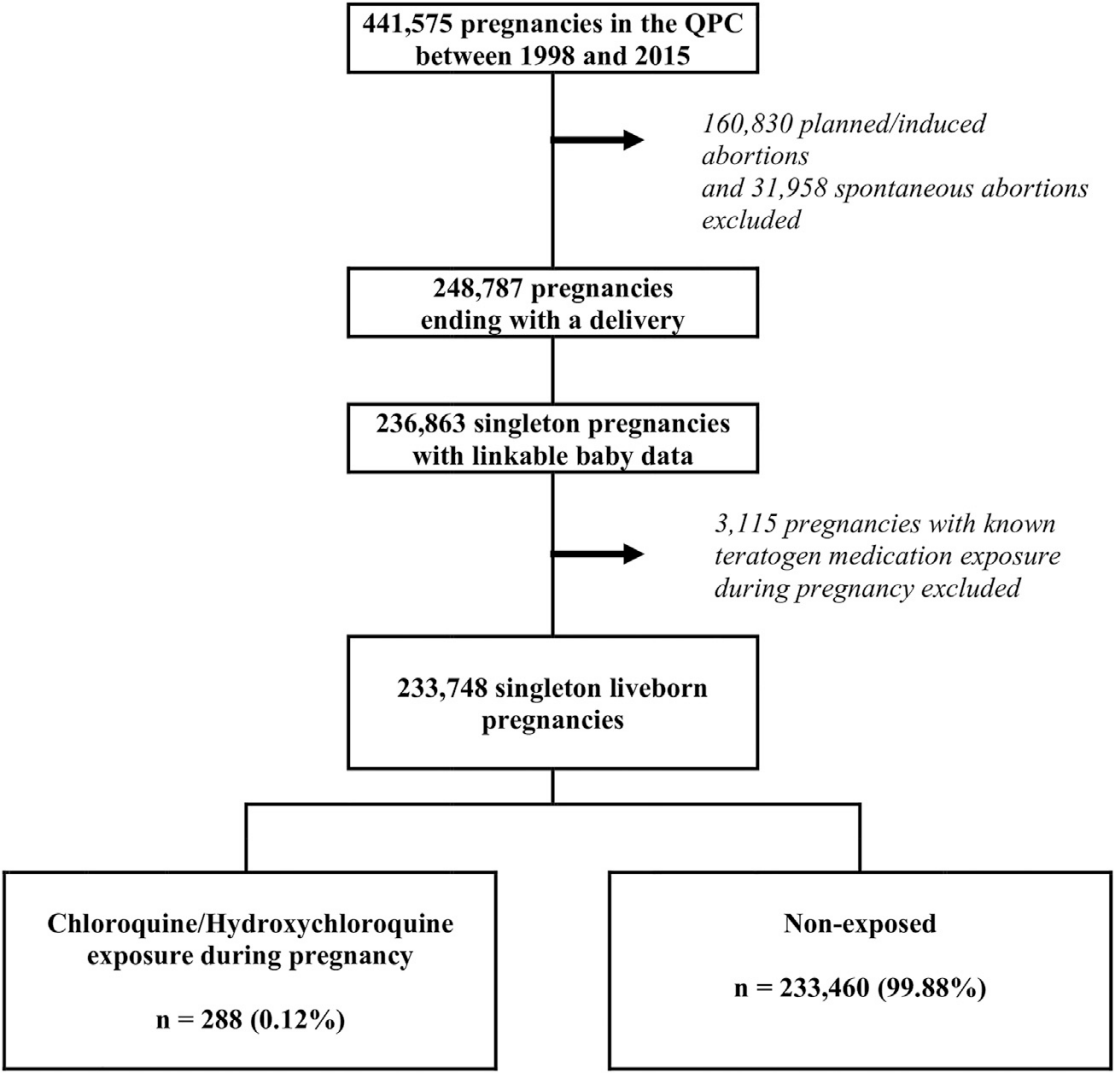
Selection of the study cohort within the Quebec Pregnancy Cohort (QPC).

Pregnant women in the QPC were eligible for this study if they were continuously covered by the Quebec Prescription Drug Insurance for ≥12 months before and during pregnancy, and had given birth to liveborn singletons. This was done because twin pregnancies are at increased risk of adverse pregnancy outcomes regardless of gestational medication exposures. We also excluded pregnancies exposed to known teratogens as described by Kulaga et al. (2009), and those resulting in minor malformations alone or chromosomal abnormalities in the newborns (Figure 1). Minor malformations are selectively identified and do not reflect the true prevalence; chromosomal abnormalities are not related to medication use.

The study was approved by the Sainte-Justine’s Hospital Ethics Committee. The Quebec “Commission d’accès à l’information” authorized database linkages.

### Chloroquine or Hydroxychloroquine Exposure

We identified CQ and HCQ prescriptions (Supplementary Table S1) filled from the Quebec Prescription Drug Insurance database, using timing of exposure determined by the dispensed date and duration of treatment. The relevant exposure time-window for the analyses of malformations was the first trimester (0–14 completed weeks of gestation since LMP; organogenesis) (Bérard et al., 2016; Howley et al., 2016); the relevant exposure time-window was the second/third trimester of pregnancy (>14 weeks of gestation until delivery) for the analyses of prematurity and LBW (intra-uterine growth). Pregnancies were considered as exposed if women had filled at least one CQ or HCQ prescription during the relevant time-windows or if they had filled a prescription with a duration that overlapped the relevant time-window (to consider CQ/HCQ half-life). The comparator group was defined as pregnancies with no exposure to CQ/HCQ during the time-windows of interest. Exposure to other anti-malarials (Supplementary Table S2) were used as an active comparator to take into account potential indication bias. Indication bias was also considered by adjusting for diagnoses of malaria, and diagnoses of SLE, and RA or use of other anti-rheumatic drugs in the analyses.

Duration of use was defined as the number of days with active prescription fillings, and the defined daily dose was defined as a CQ equivalent dosage divided by the duration of use (Liu et al., 2020).

Data on prescription fillings have been validated and compared to maternal reports in the QPC; the positive predictive value (PPV) of prescription drug data was ≥87% (95%CI: 70–100%) and the negative predictive value (NPV) was ≥92% (95%CI: 86–98%) (Zhao et al., 2017).

### Outcomes

Cases of prematurity were identified from the RAMQ and MedEcho databases, and defined as delivery at less than 37 weeks gestation.

Cases of LBW were identified from the ISQ database, and defined as a newborn of less than 2,500 g.

Cases of MCM diagnosed in the first 12 months of life were identified from the RAMQ and MedEcho databases and defined according to ICD-9 and ICD-10 codes (Supplementary Table S3), which have been validated against patient charts with high PPV (78.1%) and NPV (94.2%) (Blais et al., 2013). All organ systems were considered and high PPV (over 80%) have also been reported for specific MCM (Blais et al., 2013). Twelve months after birth was needed to allow for late detection, and validation of early diagnoses.

### Statistical Analyses

Within the identified study cohort, we conducted 3 case-control analyses to quantify the effect of CQ/HCQ exposure during pregnancy on the occurrence of prematurity, LBW, and MCM. All pregnancies were considered and no selection of controls was done.

Potential confounders considered for all analyses were: 1) sociodemographic variables measured at the time of LMP including maternal age, receipt of social assistance during or 1 year before pregnancy (yes/no), area of residence (urban/rural); 2) maternal chronic comorbidities (12 months pre-pregnancy and during first half of pregnancy) including hypertension, diabetes, asthma, epilepsy, depression, thyroid disorders, obesity, as well as the diagnoses of malaria, RA, and SLE to adjust for potential indication bias (see Supplementary Table S4 for codes used); 3) Health care utilization including hospitalizations or emergency department visits (yes/no), number of general practitioner visits and specialist visits (12 months pre-pregnancy); 4) lifestyle variables: tobacco and alcohol dependence (Supplementary Table S4); 5) Pregnancy related variables including folic acid use [prescribed high dose (>5 mg/d) and prescribed over-the-counter (OTC) dosage only] in the 6-months prior to LMP and during first trimester (Supplementary Table S4), and previous pregnancy (spontaneous or planned abortion, delivery) in the year prior to LMP (yes/no). We also considered whether pregnant women were followed by an obstetrician/gynaecologist (yes/no), and if other medications were used during pregnancy (besides CQ/HCQ and medication used to identify comorbidities), including HIV drugs (Supplementary Table S4).

The unit of analysis was the pregnancy. Means and proportions for continuous and dichotomous variables were calculated, respectively. Crude and adjusted odds ratios (aOR) with 95% confidence intervals (95%CI) were calculated for each outcome separately. Multivariable generalized estimating equations were used to estimate the association between CQ/HCQ and prematurity, LBW, and MCM risks, independently, accounting for clustering by family (mother). All statistical analyses were performed using SAS (SAS Institute Inc., Version 9.2, Cary, NC, United States).

## Results

Of the 441,514 pregnancies within the QPC, 233,748 met inclusion criteria and were considered for analyses; 288 were exposed to CQ/HCQ at any time during pregnancy (comprising 727 prescriptions filled) (Figure 1; Table 1), with the majority during the first trimester (*n*, 218). HCQ was more frequently prescribed [524 prescriptions filled (72.1%) vs. 203 (27.9%) for CQ, with 89 pregnancies exposed to both CQ and HCQ, data not shown]. We identified that 183 (63.5%) pregnancies were exposed to CQ; and 105 (36,5%) were exposed to HCQ (Supplementary Table S5). On average, pregnant users were exposed for 42 days during the first trimester [standard-deviation (SD), 27.6] (mean of 1.7 prescriptions filled), and 72 days (SD, 52) (mean of 2.7 prescriptions filled) during the second/third trimesters (Supplementary Table S5). Mean daily dosages were similar in the first and second half of pregnancy (218.4 mg CQ equivalent dosage (SD, 154.6) in the first trimester and (215.6 mg CQ equivalent dosage (SD, 157.7) in the second/third trimester (Supplementary Table S5). CQ/HCQ was used for RA (17.4%), SLE (16.3%) or malaria (0.7%) (Table 1). CQ/HCQ users were slightly older; of higher socio-economic status (83.7% adherent vs. 77.1% in non-users); more likely to use high dose (>5 mg/d) folic acid, although the overall prevalence remained low; more likely to have hypertension, diabetes or asthma; and had a higher prevalence of health services utilization including other medication use (Table 1).

**TABLE 1:** Characteristics of the pregnancies based on chloroquine (CQ)/hydroxychloroquine (HCQ) exposure status.

	CQ/HCQ exposure during pregnancy			
Characteristic	All pregnancies *n* = 233,748	Yes *n* = 288	No *n* = 233,460	*p*-value
Other study drug exposure – n (%):				
Other antimalarial medications^a^	151 (0.06)	2 (0.69)	149 (0.06)	<0.0001
Maternal characteristics on 1st day of gestation:				
Maternal age on 1DG^b^ (years) – mean ± sd	28.22 ± 5.59	29.72 ± 5.27	28.22 ± 5.59	<0.0001
Rx insurance adherent – n (%)	180,337 (77.15)	241 (83.68)	180,096 (77.14)	0.008
Urban dweller – n (%)	192,480 (82.35)	236 (81.94)	192,244 (82.35)	0.86
Baby characteristics at birth:				
Gestational age (weeks) – mean ± sd	38.85 ± 1.80	38.70 ± 1.97	38.85 ± 1.80	0.20
Birth weight (grams) – mean ± sd	3,348.96 ± 545.17	3,285.2 ± 576.5	3,349.0 ± 545.1	0.047
Male gender – n (%)	120,157 (50.69)	146 (50.69)	120,011 (51.41)	0.81
Health care utilization in the 12-months prior to the 1DG:				
Emergency visit and/or hospitalization– n (%)	79,500 (34.01)	104 (36.11)	79,393 (34.01)	0.45
General practitioner (GP) visits – n (%)				
0	49,439 (21.15)	50 (17.36)	9,389 (21.16)	
1–3	88,465 (37.85)	105 (36.46)	88,360 (37.85)	
4 or more	95,844 (41.00)	133 (46.18)	95,711 (41.00)	0.136
Number of specialists visits – n (%)				
0	91,733 (39.24)	65 (22.57)	91,668 (39.26)	
1–2	58,199 (24.90)	68 (23.61)	58,131 (24.90)	
3 or more	83,816 (35.86)	155 (53.82)	83,661 (35.84)	<0.0001
Other prescribed medications^c^ - n (%)				
0	69,649 (29.80)	31 (10.76)	69,618 (29.82)	
1–2	79,998 (34.22)	80 (27.78)	79,918 (34.23)	
3 or more	84,101 (35.98)	177 (61.46)	84,924 (35.95)	<0.0001
At least one diagnosis in the 6-months prior to 1DG until the end of pregnancy:				
SLE^d^ – n (%)	175 (0.07)	47 (16.32)	128 (0.05)	<0.0001
Malaria – n (%)	60 (0.03)	2 (0.69)	58 (0.02)	<0.0001
Rheumatoid arthritis - n (%)	434 (0.19)	50 (17.36)	384 (0.16)	<0.0001
HIV medications use during pregnancy^e^	342 (0.15)	2 (0.69)	340 (0.15)	<0.0001
Folic acid exposure in the 6-months prior to LMP and during first trimester - n (%)	8,772 (3.75)	37 (12.85)	8,735 (3.74)	<0.0001
Maternal co-morbidities in the 12-months prior to LMP or first trimester:	5,987 (2.56)	8 (2.78)	5,979 (2.56)	0.82
Hypertension – n (%)	5,304 (2.27)	13 (4.51)	5,291 (2.27)	0.011
Diabetes – n (%)	28,273 (12.10)	46 (15.97)	28,227 (12.09)	0.044
Asthma – n (%)	10,381 (4.44)	26 (9.03)	10,355 (4.44)	0.0002
Thyroid disorders – n (%)	7,378 (3.16)	7 (2.43)	7,371 (3.16)	0.48
Tobacco dependence – n (%)	929 (0.40)	2 (0.69)	927 (0.40)	0.42
Alcohol dependence – n (%)	2,393 (1.02)	5 (1.74)	2,388 (1.02)	0.23
Other drug dependence – n (%)				
Pregnancy follow-up by obstetrician – n (%)	133,693 (57.20)	188 (65.28)	133,505 (57.19)	0.006
Pregnancy in the year prior to the 1st day of gestation – n (%)	20,105 (8.60)	16 (5.56)	20,089 (8.60)	0.065

a Atovaquone, mefloquine, primaquine, proguanil, sulfadoxine-pyrimethamine, and halofantrine.

b First day of gestation defined as the first day of the last menstrual period (LMP).

c Other prescribed medications than the study medications and medications used to assess the maternal co-morbidities prior pregnancy.

d Systemic lupus erythematosus.

e Alone or combined: abacavir, adefovir dipivoxil, atazanavir, AZT, bictegravir, cobicistat, darunavir, delavirdine, didanosine, dolutegravir, doravirine, efavirenz, elvitegravir, emtricitabine, enfuvirtide, etravirine, fosamprenavir, indinavir, lamivudine, lopinavir, maraviroc, nelfinavir, nevirapine, raltegravir, retrovir, rilpivirine, ritonavir, saquinavir, stavudine, tenofovir disoproxil, tenofovir alafenamide, tipranavir, and zidovudine.

### Prematurity

Within the study population, 15,676 (6.7%) pregnancies resulted in a premature delivery (Table 2). Adjusting for potential confounders including indication for use or use of other-antimalarials, CQ/HCQ use during the second or third trimester, which is the relevant time-window for these analyses, was not statistically significantly associated with the risk of prematurity (aOR 0.87, 95%CI 0.46–1.67; 15 exposed cases) (Table 2). Exposure to other antimalarials during the same period had a similar association with the risk of prematurity (aOR 0.77, 95%CI 0.23–2.53; 3 exposed cases).

**TABLE 2:** Association between CQ/HCQ exposure during pregnancy and the risk of prematurity.

	Prematurity (<37 weeks of gestation)			
Variables	Yes *n* = 15,676	No *n* = 218,072	Crude OR (95% CI)	Adjusted OR (95% CI)
	n (%)			
Study medication exposures:				
During the 1st trimester:				
CQ/HCQ	23 (0.15)	195 (0.09)	1.62 (1.03–2.53)	1.39 (0.84–2.30)
Other antimalarial medications^a^	10 (0.06)	119 (0.05)	1.21 (0.57–2.57)	1.22 (0.59–2.54)
During the 2nd/3rd trimesters:				
CQ/HCQ	15 (0.10)	144 (0.07)	1.13 (0.65–1.96)	0.87 (0.46–1.67)
Other antimalarial medications^a^	3 (0.02)	53 (0.02)	0.76 (0.23–2.59)	0.77 (0.23–2.53)
Maternal age at the 1DG^b^ - mean (SD)	28.11 ± 5.91	28.23 ± 5.57	1.00 (0.99–1.00)	1.00 (1.00–1.00)
Adherent vs. welfare recipient	10,917 (69.64)	169,420 (77.69)	0.68 (0.65–0.70)	0.73 (0.70–0.76)
Urban dweller	12,924 (82.44)	179,556 (82.34)	1.01 (0.97–1.05)	0.96 (0.92–1.01)
Diagnosis during pregnancy for:				
SLE^c^	25 (0.16)	150 (0.07)	2.39 (1.56–3.66)	1.95 (1.23–3.07)
Malaria	5 (0.03)	55 (0.03)	1.38 (0.58–3.30)	1.22 (0.50–2.96)
Rheumatoid arthritis	29 (0.18)	405 (0.19)	1.00 (0.68–1.46)	0.83 (0.57–1.21)
Comorbidities in the year prior to the 1DG:				
Hypertension	666 (4.25)	5,321 (2.44)	1.66 (1.53–1.81)	1.44 (1.32–1.57)
Diabetes	620 (3.96)	4,684 (2.15)	1.80 (1.64–1.97)	1.53 (1.40–1.67)
Asthma	2,273 (14.50)	26,000 (11.92)	1.21 (1.16–1.27)	1.05 (1.00–1.10)
Thyroid disorders	744 (4.75)	9,637 (4.42)	1.07 (0.99–1.16)	0.97 (0.90–1.05)
Diagnosis of dependence to:				
Tobacco	851 (5.43)	6,527 (2.99)	1.77 (1.64–1.91)	1.53 (1.41–1.65)
Alcohol	124 (0.79)	805 (0.37)	2.04 (1.67–2.48)	1.11 (0.90–1.37)
Other drugs	372 (2.37)	2,021 (0.93)	2.44 (2.17–2.74)	1.82 (1.61–2.06)
HIV drug^d^ use during the 1st trimester	32 (0.20)	174 (0.08)	1.70 (0.86–3.36)	1.59 (0.80–3.15)
HIV drug use during the 2nd/3rd trimesters:	45 (0.29)	288 (0.13)	1.51 (0.84–2.72)	1.22 (0.67–2.20)
In the year prior to the 1DG:				
Emergency visit or hospitalization	6,143 (39.19)	73,357 (33.64)	1.24 (1.20–1.28)	1.07 (1.03–1.11)
General practitioner visit				
0	2,980 (19.01)	46,459 (21.30)	Ref.	Ref.
1–3	5,521 (35.22)	82,944 (38.04)	1.03 (0.99–1.08)	0.99 (0.95–1.04)
4 or more	7,175 (45.77)	88,669 (40.66)	1.22 (1.17–1.28)	1.05 (1.00–1.11)
Specialist visits				
0	5,539 35.33)	86,194 (39.53)	Ref.	Ref.
1–2	3,652 (23.30)	54,547 (25.01)	1.04 (0.99–1.08)	0.98 (0.93–1.02)
3 or more	6,485 (41.37)	77,331 (35.46)	1.27 (1.23–1.32)	1.08 (1.03–1.13)
Other prescribed medications^e^				
0	4,003 (25.54)	65,646 (30.10)	Ref.	Ref.
1–2	5,072 (32.36)	74,926 (34.36)	1.11 (1.07–1.16)	1.07 (1.03–1.12)
3 or more	6,601 (42.11)	77,500 (35.54)	1.36 (1.30–1.41)	1.14 (1.09–1.20)
Pregnancy follow-up by obstetrician	9,585 (61.14)	124,108 (56.91)	1.19 (1.15–1.24)	1.19 (1.14–1.23)
Pregnancy in the year prior the 1DG	1,495 (9.54)	18,610 (8.53)	1.12 (1.06–1.18)	0.98 (0.93–1.04)
Folic acid consumption before the end of the 1st trimester	772 (4.92)	8,000 (3.67)	1.33 (1.23–1.44)	1.16 (1.07–1.26)

a Atovaquone, mefloquine, primaquine, proguanil, sulfadoxine-pyrimethamine, and halofantrine.

b First day of gestation defined as the first day of the last menstrual period (LMP).

c Systemic lupus erythematosus.

d Alone or combined: abacavir, adefovir dipivoxil, atazanavir, AZT, bictegravir, cobicistat, darunavir, delavirdine, didanosine, dolutegravir, doravirine, efavirenz, elvitegravir, emtricitabine, enfuvirtide, etravirine, fosamprenavir, indinavir, lamivudine, lopinavir, maraviroc, nelfinavir, nevirapine, raltegravir, retrovir, rilpivirine, ritonavir, saquinavir, stavudine, tenofovir disoproxil, tenofovir alafenamide, tipranavir, and zidovudine.

e Other prescribed medications than the study medications and medications used to assess the maternal co-morbidities prior pregnancy.

### LBW

Within the study population, 11,866 (5.1%) pregnancies resulted in a LBW newborn (Table 3). Adjusting for potential confounders including indication for use, and use of other-antimalarials, CQ/HCQ use during the second or third trimester, which is the relevant time-window for these analyses, was not statistically significantly associated with the risk of LBW (aOR 0.97, 95%CI 0.46–2.08; 14 exposed cases) (Table 3).

**TABLE 3:** Association between CQ/HCQ exposure during pregnancy and the risk of LBW.

	Low birth weight (<2,500 g)			
Variables	Yes *n* = 11,866	No *n* = 221,882	Crude OR (95% CI)	Adjusted OR (95% CI)
	n (%)			
Study medication exposures:				
During the 1st trimester:				
CQ/HCQ	18 (0.15)	200 (0.09)	1.46 (0.87–2.44)	1.11 (0.59–2.06)
Other antimalarial medications^a^	5 (0.04)	124 0.06)	0.89 (0.37–2.16)	0.89 (0.36–2.17)
During the 2nd/3rd trimesters:				
CQ/HCQ	14 (0.12)	145 (0.07)	1.45 (0.80–2.65)	0.97 (0.46–2.08)
Other antimalarial medications^a^	1 (0.01)	55 (0.02)	0.37 (0.08–1.81)	0.37 (0.07–1.88)
Maternal age at the 1DG^b^ - mean (SD)	28.16 ± 5.99	28.23 ± 5.57	1.00 (1.00–1.03)	1.00 (1.00–1.01)
Adherent vs. welfare recipient	8,016 (67.55)	172,321 (77.66)	0.61 (0.59–0.64)	0.68 (0.65–0.71)
Urban dweller	9,785 (82.46)	182,695 (82.34)	1.01 (0.96–106)	0.95 (0.90–1.00)
Diagnosis during pregnancy for:				
SLE^c^	27 (0.23)	148 (0.07)	3.35 (2.17–5.19)	2.80 (1.71–4.59)
Malaria	3 (0.03)	57 (0.03)	0.96 (0.28–3.36)	0.87 (0.24–3.11)
Rheumatoid arthritis	27 (0.23)	407 (0.18)	1.24 (0.85–1.83)	1.05 (0.72–1.54)
Comorbidities in the year prior to the 1DG:				
Hypertension	524 (4.42)	5,463 (2.46)	1.70 (1.54–1.87)	1.52 (1.38–1.67)
Diabetes	336 (2.83)	4,968 (2.24)	1.26 (1.12–1.41)	1.04 (0.93–1.17)
Asthma	1,881 (15.85)	26,392 (11.89)	1.35 (1.28–1.43)	1.15 (1.09–1.22)
Thyroid disorders	541 (4.56)	9,840 (4.43)	1.02 (0.94–1.12)	0.96 (0.88–1.05)
Diagnosis of dependence to:				
Tobacco	868 (7.32)	6,510 (2.93)	2.47 (2.29–2.67)	2.09 (1.93–2.27)
Alcohol	124 (1.05)	805 (0.36)	2.77 (2.28–3.36)	1.24 (1.00–1.53)
Other drugs	348 (2.93)	2,045 (0.92)	3.03 (2.68–3.42)	2.02 (1.78–2.31)
HIV drug^d^ use during the 1st trimester	32 (0.27)	174 (0.08)	1.65 (0.83–3.30)	1.54 (0.77–3.09)
HIV drug use during the 2nd/3rd trimesters	46 (0.39)	287 (0.13)	2.18 (1.21–3.95)	1.68 (0.93–3.06)
In the year prior to the 1DG:				
Emergency visit or hospitalization	4,450 (37.50)	75,050 (33.82)	1.15 (1.11–1.20)	0.99 (0.95–1.04)
General practitioner visit				
0	2,336 (19.69)	47,103 (21.23)	Ref.	Ref.
1–3	4,168 (35.13)	84,297 (37.99)	0.99 (0.95–1.05)	0.97 (0.92–1.03)
4 or more	5,362 (45.19)	90,482 (40.78)	1.17 (1.11–1.23)	1.02 (0.96–1.08)
Specialist visits				
0	4,369 (36.82)	87,364 (39.37)	Ref.	Ref.
1–2	2,763 (23.29)	55,436 (24.98)	0.99 (0.95–1.04)	0.94 (0.89–0.98)
3 or more	4,734 (39.90)	79,082 (35.64)	1.18 (1.13–1.23)	1.00 (0.95–1.05)
Other prescribed medications^e^				
0	3,079 (25.95)	66,570 (30.00)	Ref.	Ref.
1–2	3,731 (31.44)	76,267 (34.37)	1.06 (1.01–1.11)	1.03 (0.98–1.08)
3 or more	5,056 (42.61)	79,045 (35.62)	1.35 (1.29–1.42)	1.15 (1.09–1.21)
Pregnancy follow-up by obstetrician	7,424 (62.57)	126,269 (56.91)	1.27 (1.22–1.32)	1.29 (1.23–1.34)
Pregnancy in the year prior the 1DG	1,140 (9.61)	18,965 (8.55)	1.12 (1.05–1.20)	1.04 (0.97–1.11)
Folic acid consumption before the end of the 1st trimester	610 (5.14)	8,162 (3.68)	1.41 (1.29–1.53)	1.27 (1.16–1.38)

a Atovaquone, mefloquine, primaquine, proguanil, sulfadoxine-pyrimethamine, and halofantrine.

b First day of gestation defined as the first day of the last menstrual period (LMP).

c Systemic lupus erythematosus.

d Alone or combined: abacavir, adefovir dipivoxil, atazanavir, AZT, bictegravir, cobicistat, darunavir, delavirdine, didanosine, dolutegravir, doravirine, efavirenz, elvitegravir, emtricitabine, enfuvirtide, etravirine, fosamprenavir, indinavir, lamivudine, lopinavir, maraviroc, nelfinavir, nevirapine, raltegravir, retrovir, rilpivirine, ritonavir, saquinavir, stavudine, tenofovir disoproxil, tenofovir alafenamide, tipranavir, and zidovudine.

e Other prescribed medications than the study medications and medications used to assess the maternal co-morbidities prior pregnancy.

### Major Malformations

Within the study population, 25,351 pregnancies resulted in a newborn with MCM (Table 4). Adjusting for potential confounders including indication for use, and use of other-antimalarials, CQ/HCQ use during the first trimester, which is the relevant time-window for organogenesis, was not statistically significantly associated with the risk of MCM (aOR 1.01, 95%CI 0.67–1.52; 26 exposed cases) (Table 4). Exposure to other antimalarials during the same period had a similar association with the risk of MCM.

**TABLE 4:** Association between CQ/HCQ exposure during the first trimester and the risk of overall major congenital malformations.

	Major congenital malformation			
Variables	Yes *n* = 25,351	No *n* = 208,397	Crude OR (95% CI)	Adjusted OR (95% CI)
	n (%)			
Study medication exposures during the 1st trimester:				
CQ/HCQ	26 (0.10)	192 (0.09)	117 (0.06)	1.01 (0.67–1.52)
Other antimalarial medications^a^	12 (0.05)	1.11 (0.74–1.67)	0.84 (0.47–1.52)	0.84 (0.47–1.51)
Maternal age at the 1DG^b^ - mean (SD)	28.08 ± 5.57	28.24 ± 5.59	0.99 (0.99–1.00)	0.99 (0.99–1.00)
Adherent vs. welfare recipient	19,479 (76.84)	160,858 (77.19)	0.98 (0.95–1.01)	1.02 (0.99–1.05)
Urban dweller	21,888 (83.24)	171,392 (82.18)	1.07 (1.03–1.11)	1.05 (1.02–1.09)
Diagnosis during pregnancy for:				
SLE^c^	16 (0.06)	159 (0.08)	0.82 (0.50–1.36)	0.73 (0.44–1.20)
Malaria	4 (0.02)	56 (0.03)	0.59 (0.21–1.62)	0.54 (0.20–1.50)
Rheumatoid arthritis	70 (0.28)	364 (0.17)	1.58 (1.22–2.05)	1.49 (1.14–1.94)
Comorbidities^b^				
Hypertension	736 (2.90)	5,251 (2.52)	1.15 (1.06–1.25)	1.08 (1.00–1.17)
Diabetes	667 (2.63)	4,637 (2.23)	1.18 (1.09–1.29)	1.10 (1.01–1.20)
Asthma	3,343 (13.19)	24,930 (11.96)	1.12 (1.07–1.16)	1.06 (1.01–1.10)
Thyroid disorders	1,223 (4.82)	9,158 (4.39)	1.10 (1.04–1.17)	1.06 (1.00–1.13)
Diagnosis of dependence to:				
Tobacco	879 (3.47)	6,499 (3.12)	1.11 (1.04–1.20)	1.09 (1.01–1.17)
Alcohol	111 (0.44)	8,185 (0.39)	1.12 (0.92–1.37)	0.99 (0.81–1.22)
Other drugs	304 (1.20)	2,089 (1.00)	1.20 (1.06–1.35)	1.14 (1.00–1.29)
HIV drug^d^ use during the 1st trimester:	34 (0.13)	172 (0.08)	1.62 (1.10–2.38)	1.51 (1.02–2.23)
In the year prior to the 1DG:				
Emergency visit or hospitalization	9,004 (35.52)	70,496 (33.83)	1.08 (1.05–1.11)	1.00 (0.97–1.03)
General practitioner visit				
0	4,918 (19.40)	44,521 (21.36)	Ref.	Ref.
1–3	9,469 (37.35)	78,996 (37.91)	1.08 (1.05–1.12)	1.01 (0.98–1.05)
4 or more	10,964 (43.25)	84,880 (40.73)	1.17 (1.13–1.21)	1.04 (1.01–1.08)
Specialist visits				
0	9,229 (36.40)	82,504 (39.59)	Ref.	Ref.
1–2	6,419 (25.32)	51,780 (24.85)	1.11 (1.07–1.15)	1.06 (1.02–1.10)
3 or more	9,703 (38.27)	74,113 (35.56)	1.17 (1.13–1.20)	1.10 (1.06–1.15)
Other prescribed medications^e^				
0	7,115 (28.07)	62,534 (30.01)	Ref.	Ref.
1–2	8,508 (33.56)	71,490 (34.30)	1.05 (1.01–1.08)	1.07 (1.03–1.11)
3 or more	9,728 (38.37)	74,373 (35.69)	1.15 (1.11–1.19)	1.09 (1.05–1.13)
Pregnancy follow-up by obstetrician	15,304 (60.37)	118,389 (56.81)	1.16 (1.13–1.19)	1.15 (0.90–0.99)
Pregnancy in the year prior to 1DG	2,188 (8.63)	17,917 (8.60)	1.00 (0.96–1.05)	0.94 (1.00–1.15)
Folic acid consumption before the end of the 1st trimester	1,051 (4.15)	7,721 (3.70)	1.12 (1.05–1.20)	1.07

a Atovaquone, mefloquine, primaquine, proguanil, sulfadoxine-pyrimethamine, and halofantrine.

b First day of gestation defined as the first day of the last menstrual period (LMP).

c Systemic lupus erythematosus.

d Alone or combined: abacavir, adefovir dipivoxil, atazanavir, AZT, bictegravir, cobicistat, darunavir, delavirdine, didanosine, dolutegravir, doravirine, efavirenz, elvitegravir, emtricitabine, enfuvirtide, etravirine, fosamprenavir, indinavir, lamivudine, lopinavir, maraviroc, nelfinavir, nevirapine, raltegravir, retrovir, rilpivirine, ritonavir, saquinavir, stavudine, tenofovir disoproxil, tenofovir alafenamide, tipranavir, and zidovudine.

e Other prescribed medications than the study medications and medications used to assess the maternal co-morbidities prior pregnancy.

Adjusting for potential confounders, no statistically significant increase in the risk of organ specific defects were observed with first-trimester use of CQ/HCQ, or other antimalarials (Supplementary Table S6). Supplementary Table S7 describes the MCM identified with CQ/HCQ use.

## Discussion

This study was performed in one of the few large population-based pregnancy cohort to examine CQ and HCQ exposures during pregnancy, and did not show clear indications that CQ or HCQ use during pregnancy were associated with increased risk of prematurity, LBW, and MCM, adjusting for underlying conditions, and additional potential risk factors.

More than one third of the CQ/HCQ pregnant users had SLE (16.32%, 47/288), RA (17.36%, 50/288), or malaria (0.67%, 2/288). We found a non-significant 13% decrease in the risk of prematurity among those using CQ/HCQ in the second/third trimester. Our findings on prematurity are consistent with Kroese et al. (2017) who found that duration of gestation was longer amongst premature newborns exposed *in utero* to HCQ. Given our number of exposed cases, it was not possible for us to further stratify on prematurity status. However, the mean gestational age of CQ/HCQ users and non-users were similar. We found no significant association between CQ/HCQ exposure and increased risk of LBW and MCM, which is similar to previous studies (Costedoat-Chalumeau et al., 2003; Motta et al., 2005; Clowse et al., 2006; Diav-Citrin et al., 2013; Cooper et al., 2014; Kroese et al., 2017; Bermas et al., 2018; Andersson et al., 2020), but our study has adjusted for all known and measurable potential confounders for adverse pregnancy outcomes; and adjustment was also made on health services utilization, which is considered a proxy for severity of diseases. Although we have a large cohort of pregnant CQ/HCQ users, we had few exposed cases for any of the specific defects studied. Contrary to early concerns (Ingster-Moati and Albuisson, 2010; Moore and Davis, 2018), we did not identify any cases of ocular defects associated with CQ/HCQ. Of note, we only considered exposure during organogenesis for our analyses on major malformations, and caution is still warranted given our sample size. Finally, data on CQ/HCQ exposures were collected prospectively contrary to others (Cooper et al., 2014) who collected the information retrospectively after birth, which may lead to recall bias.

Our findings was also consistent with the recent study Andersson et al. (Andersson et al., 2020). Using the Danish nationwide cohort (1996–2016), Andersson et al. (2020) found that exposure to the 4-aminoquinolines drugs CQ and HCQ during pregnancy was not associated with an increased risk of MCM (prevalence OR 0.94, 95%CI 0.59–1.52; 34 exposed cases), preterm birth (prevalence OR 0.97, 95%CI 0.73–1.28; 103 exposed cases) or small for gestational age (prevalence OR 1.18, 95%CI 0.93–1.50; 165 exposed cases), compared to propensity-score–matched unexposed pregnancies. No significant associations between exposure to CQ or HCQ individually and risk of MCM, preterm birth or small for gestational age were identified. This study did not investigate the risk of specific defects.

While, using the Medicaid Analytic eXtract (MAX, 2000–2014) and IBM MarketScan Research Database (MarketScan, 2003–2015), Huybrechts et al. (2021) compared HCQ-exposed pregnancies with matched unexposed pregnancies, and found a small increase in the risk of MCM associated with first trimester HCQ use (adjusted RR 1.26, 95%CI 1.04–1.54; 112 exposed cases); it was 1.33 (95%CI 1.08–1.65) for a daily dose of >400 mg and 0.95 (95%CI 0.60–1.50) for a daily dose of <400 mg. Given data supporting the benefits of CQ/HCQ during pregnancy for malarial prophylaxis (Mejia Torres et al., 2013), and lupus pregnancy outcome (Leroux et al., 2015), CQ/HCQ should be given and available primarily to those with malaria (prevention and treatment), and rheumatic diseases.

### Strengths and Potential Limitations

Study strengths include the use of a population-based prospective pregnancy cohort with linkage of data at the individual level; this allowed for analyses on a large number of pregnancies with detailed information regarding exposure, outcomes, and potential confounders. QPC data on prescriptions filled (Zhao et al., 2017), MCM (Blais et al., 2013), gestational age (Vilain et al., 2008) and birth weight (Vilain et al., 2008) have been validated. We have adjusted for all known and measurable potential confounding variables for unfavorable pregnancy outcomes, including maternal chronic comorbidities, indications for study medication uses, lifestyles variables including the use of alcohol, tobacco, illicit substances, and high dose folic acid; adjustment was also made on health care utilization, which is considered as a proxy for diseases severity (Bérard et al., 2021).

One potential limitation is missing information on potential confounders such as smoking, alcohol, and over the counter folic acid use. Nevertheless, tobacco and alcohol dependence, and prescribed folic acid were used as proxies for these variables. The majority of folic acid users were on high dose (>5 mg/d), and thus this was likely a proxy for high-risk pregnancy as well in our study. The identification of MCM was based on diagnosis codes that had been recorded in claims and hospital databases. If misclassification of exposure would be present, it would bias the results of our study toward the null excepting that a higher proportion of malformation diagnoses were identified in pregnancies of mothers with SLE or RA. In spite of the fact that we have shown a high positive predictive value for malformations (Zhao et al., 2017), the potential for some misclassification still exists. We used filled prescriptions and not actual intake, but Zhao et al. (2017) have shown that prescription fillings data in the QPC were valid when compared to maternal report. Indeed, given that we have prescription fillings data, we wanted to ensure that women took their medications (intake). Patient charts are not good for this because they are providing medication prescribing and not intake. Hence, we have compared pregnant women’s self-report of medications use (any medications in real-time to minimize recall bias) to the data on medication fillings (claims database used to put in place the QPC) on a sample of women included in the QPC (Zhao et al., 2017). Given that we consider self-report the “gold standard,” we have calculated the PPV and NPV for the use of the QPC data on medications. Both the PPV and NPV for medication use were high within the QPC. Because we only included pregnant women covered by the Quebec prescription drug insurance plan, generalizability of results to those covered by private medication insurance could be affected (Bérard et al., 2021). However, validation studies have shown that publicly insured pregnant women have similar characteristics and co-morbidities with those who have private drug insurance plans (Bérard and Lacasse, 2009). Our estimates could be slightly upwards biased because we only included deliveries in our analyses as is done in most studies on medications and pregnancy. Despite the fact that health care utilization has been adjusted for and considered as a proxy for disease severity, residual confounding from severity of disease could continue to exist. Given that our medication definitions are dichotomous, considering second/third trimester in a combined manner minimizes immortal time bias. Furthermore, prematurity prevalence is 5–7%, rendering immortal time bias very unlikely. Finally, the MCM prevalence of 8.6% is higher than what is routinely reported (3–5%) (Egbe, 2015). This could be partly explained by the Founders’ effect in the province of Quebec (Laberge, 2007; Zhao et al., 2015). It can also be partly explained by the fact that we have included all pregnancies between 1998 and 2015. Even though the baseline prevalence of MCMs is high, it does not differ among comparison groups, and thus does not invalidate our findings. This, however, could limit the generalizability of our results.

## Conclusion

In this large cohort of exposed pregnancies, maternal exposure to CQ/HCQ during pregnancy has not been shown to increase the risk of prematurity, LBW, or MCM. These findings are consistent with other studies in the literature, and are reassuring. However, given that CQ/HCQ can cause or worsen heart arrhythmia, concerns remain for use in those with pre-existing heart conditions (Roden et al., 2020). CQ/HCQ should be given and available primarily to those with malaria (prevention and treatment), and rheumatic diseases.

## Data Availability

The data used for this study are confidential and we are forbidden to release them under our ethics committee authorization and the authorization of the Commission d'accès à l'information. Hence, the data cannot be released.

## References

[B1] Andersson N. W. Skov L. Andersen J. T. (2020). Fetal Safety of Chloroquine and Hydroxychloroquine Use during Pregnancy: a Nationwide Cohort Study. Rheumatology (Oxford) 60, 2317–2326. 10.1093/rheumatology/keaa592 33232466

[B2] Bérard A. Lacasse A. (2009). Validity of Perinatal Pharmacoepidemiologic Studies Using Data from the RAMQ Administrative Database. Can. J. Clin. Pharmacol. 16, e360–9. 19553702

[B3] Bérard A. Sheehy O. Kurzinger M.-L. Juhaeri J. (2016). Intranasal Triamcinolone Use during Pregnancy and the Risk of Adverse Pregnancy Outcomes. J. Allergy Clin. Immunol. 138, 97–104. 10.1016/j.jaci.2016.01.021 27045580

[B4] Bérard A. Sheehy O. (2014). The Quebec Pregnancy Cohort - Prevalence of Medication Use during Gestation and Pregnancy Outcomes. PLoS One 9, e93870. 10.1371/journal.pone.0093870 24705674 PMC3976411

[B5] Bérard A. Sheehy O. Zhao J.-P. Vinet E. Quach C. Kassai B. (2021). Available Medications Used as Potential Therapeutics for COVID-19: What Are the Known Safety Profiles in Pregnancy. PLoS One 16, e0251746. 10.1371/journal.pone.0251746 34010282 PMC8133446

[B6] Bermas B. L. Kim S. C. Huybrechts K. Mogun H. Hernandez-Diaz S. Bateman B. T. (2018). Trends in Use of Hydroxychloroquine during Pregnancy in Systemic Lupus Erythematosus Patients from 2001 to 2015. Lupus 27, 1012–1017. 10.1177/0961203317749046 29301469

[B7] Blais L. Bérard A. Kettani F.-Z. Forget A. (2013). Validity of Congenital Malformation Diagnostic Codes Recorded in Québec's Administrative Databases. Pharmacoepidemiol. Drug Saf. 22, 881–889. 10.1002/pds.3446 23616437

[B8] Buchanan N. M. Toubi E. Khamashta M. A. Lima F. Kerslake S. Hughes G. R. (1996). Hydroxychloroquine and Lupus Pregnancy: Review of a Series of 36 Cases. Ann. Rheum. Dis. 55, 486–488. 10.1136/ard.55.7.486 8774170 PMC1010215

[B9] Chen C.-Y. Wang F.-L. Lin C.-C. (2006). Chronic Hydroxychloroquine Use Associated with QT Prolongation and Refractory Ventricular Arrhythmia. Clin. Toxicol. 44, 173–175. 10.1080/15563650500514558 16615675

[B10] Chen J. Liu D. Liu L. Liu P. Xu Q. Xia L. (2020). A Pilot Study of Hydroxychloroquine in Treatment of Patients with Moderate COVID-19, Zhejiang Da Xue Xue Bao Yi Xue Ban. 49, 215–219. 10.3785/j.issn.1008-9292.2020.03.03 32391667 PMC8800713

[B11] Clowse M. E. B. Magder L. Witter F. Petri M. (2006). Hydroxychloroquine in Lupus Pregnancy. Arthritis Rheum. 54, 3640–3647. 10.1002/art.22159 17075810

[B12] Cooper W. O. Cheetham T. C. Li D.-K. Stein C. M. Callahan S. T. Morgan T. M. (2014). Brief Report: Risk of Adverse Fetal Outcomes Associated with Immunosuppressive Medications for Chronic Immune-Mediated Diseases in Pregnancy. Arthritis Rheumatol. 66, 444–450. 10.1002/art.38262 24504818 PMC4077326

[B13] Cortegiani A. Ingoglia G. Ippolito M. Giarratano A. Einav S. (2020). A Systematic Review on the Efficacy and Safety of Chloroquine for the Treatment of COVID-19. J. Crit. Care 57, 279–283. 10.1016/j.jcrc.2020.03.005 32173110 PMC7270792

[B14] Costedoat-Chalumeau N. Amoura Z. Duhaut P. Huong D. L. T. Sebbough D. Wechsler B. (2003). Safety of Hydroxychloroquine in Pregnant Patients with Connective Tissue Diseases: a Study of One Hundred Thirty-Three Cases Compared with a Control Group. Arthritis Rheum. 48, 3207–3211. 10.1002/art.11304 14613284

[B15] Diav-Citrin O. Blyakhman S. Shechtman S. Ornoy A. (2013). Pregnancy Outcome Following In Utero Exposure to Hydroxychloroquine: a Prospective Comparative Observational Study. Reprod. Toxicol. 39, 58–62. 10.1016/j.reprotox.2013.04.005 23602891

[B16] Egbe A. C. (2015). Birth Defects in the Newborn Population: Race and Ethnicity. Pediatr. Neonatal. 56, 183–188. 10.1016/j.pedneo.2014.10.002 25544042

[B17] Emergency Use Authorization Chloroquine Phosphate Health Care Provider Fact Sheet (2020). Emergency Use Authorization Chloroquine Phosphate Health Care Provider Fact Sheet, version date 4/3/2020. Availableat: https://www.fda.gov/media/136535/download.

[B19] Gaffar R. Pineau C. A. Bernatsky S. Scott S. Vinet É. (2019). Risk of Ocular Anomalies in Children Exposed In Utero to Antimalarials: A Systematic Literature Review. Arthritis Care Res. 71, 1606–1610. 10.1002/acr.23808 30418703

[B20] Gautret P. Lagier J.-C. Parola P. Hoang V. T. Meddeb L. Mailhe M. (2020). Hydroxychloroquine and Azithromycin as a Treatment of COVID-19: Results of an Open-Label Non-randomized Clinical Trial. Int. J. Antimicrob. Agents 56, 105949. 10.1016/j.ijantimicag.2020.105949 32205204 PMC7102549

[B21] Howley M. M. Carter T. C. Browne M. L. Romitti P. A. Cunniff C. M. Druschel C. M. (2016). Fluconazole Use and Birth Defects in the National Birth Defects Prevention Study. Am. J. Obstet. Gynecol. 214, e1–657. 10.1016/j.ajog.2015.11.022 PMC10041360 26640069

[B22] Huybrechts K. F. Bateman B. T. Zhu Y. Straub L. Mogun H. Kim S. C. (2021). Hydroxychloroquine Early in Pregnancy and Risk of Birth Defects. Am. J. Obstet. Gynecol. 224, e1–290. 10.1016/j.ajog.2020.09.007e1 PMC7501839 32961123

[B23] Ingster-Moati I. Albuisson E. (2010). Visual Neurophysiological Dysfunction in Infants Exposed to Hydroxychloroquine In Utero. Acta Paediatr. 99, 4. 10.1111/j.1651-2227.2009.01523.x 19895609

[B24] Kaplan Y. C. Ozsarfati J. Nickel C. Koren G. (2016). Reproductive Outcomes Following Hydroxychloroquine Use for Autoimmune Diseases: a Systematic Review and Meta-Analysis. Br. J. Clin. Pharmacol. 81, 835–848. 10.1111/bcp.12872 26700396 PMC4834589

[B25] Kroese S. J. de Hair M. J. H. Limper M. Lely A. T. van Laar J. M. Derksen R. H. W. M. (2017). Hydroxychloroquine Use in Lupus Patients during Pregnancy Is Associated with Longer Pregnancy Duration in Preterm Births. J. Immunol. Res. 2017, 1–5. 10.1155/2017/2810202 PMC5748114 29392142

[B26] Kulaga S. Zagarzadeh A. Bérard A. (2009). Prescriptions Filled during Pregnancy for Drugs with the Potential of Fetal Harm. BJOG 116, 1788–1795. 10.1111/j.1471-0528.2009.02377.x 19832828

[B27] Laberge A.-M. (2007). La prévalence et la distribution des maladies génétiques au Québec. Med. Sci. (Paris) 23, 997–1001. 10.1051/medsci/20072311997 18021714

[B28] Leroux M. Desveaux C. Parcevaux M. Julliac B. Gouyon J.-B. Dallay D. (2015). Impact of Hydroxychloroquine on Preterm Delivery and Intrauterine Growth Restriction in Pregnant Women with Systemic Lupus Erythematosus: a Descriptive Cohort Study. Lupus 24, 1384–1391. 10.1177/0961203315591027 26082465

[B29] Liu J. Cao R. Xu M. Wang X. Zhang H. Hu H. (2020). Hydroxychloroquine, a Less Toxic Derivative of Chloroquine, Is Effective in Inhibiting SARS-CoV-2 Infection *In Vitro* . Cell Discov 6, 16. 10.1038/s41421-020-0156-0 PMC7078228 32194981

[B30] Mejia Torres R. E. Zambrano J. O. N. Goldman I. Alam M. T. Diaz C. Udhayakumar V. (2013). Efficacy of Chloroquine for the Treatment of Uncomplicated Plasmodium Falciparum Malaria in Honduras. Am. J. Trop. Med. Hyg. 88, 850–854. 10.4269/ajtmh.12-0671 23458957 PMC3752747

[B31] Moore B. R. Davis T. M. E. (2018). Pharmacotherapy for the Prevention of Malaria in Pregnant Women: Currently Available Drugs and Challenges. Expert Opin. Pharmacother. 19, 1779–1796. 10.1080/14656566.2018.1526923 30289730

[B18] Motta M. Tincani A. Faden D. Zinzini E. Lojacono A. Marchesi A. (2005). Follow-up of infants exposed to hydroxychloroquine given to mothers during pregnancy and lactation. J Perinatol. 25 (2), 86–9. 10.1038/sj.jp.7211208 15496869

[B32] Pardridge W. M. Yang J. Diagne A. (1998). Chloroquine Inhibits HIV-1 Replication in Human Peripheral Blood Lymphocytes. Immunol. Lett. 64, 45–47. 10.1016/s0165-2478(98)00096-0 9865601

[B33] Régie de l'Assurance Maladie du Québec (2015). Rapport d'études et de statistiques. Availableat: https://www4.prod.ramq.gouv.qc.ca/IST/CD/CDF_DifsnInfoStats/CDF1_CnsulInfoStatsCNC_iut/DifsnInfoStats.aspx?ETAPE_COUR=2&LANGUE=fr-CA (Accessed July 28, 2018).

[B34] Roden D. M. Harrington R. A. Poppas A. Russo A. M. (2020). Considerations for Drug Interactions on QTc in Exploratory COVID-19 Treatment. Circulation 141, e906–e7. 10.1161/CIRCULATIONAHA.120.047521 32267732

[B35] Sammaritano L. R. Bermas B. L. Chakravarty E. E. Chambers C. Clowse M. E. B. Lockshin M. D. (2020). 2020 American College of Rheumatology Guideline for the Management of Reproductive Health in Rheumatic and Musculoskeletal Diseases. Arthritis Rheumatol. 72, 529–556. 10.1002/art.41191 32090480

[B36] Savarino A. Gennero L. Sperber K. Boelaert J. R. (2001). The Anti-HIV-1 Activity of Chloroquine. J. Clin. Virol. 20, 131–135. 10.1016/s1386-6532(00)00139-6 11166661

[B37] Schrezenmeier E. Dörner T. (2020). Mechanisms of Action of Hydroxychloroquine and Chloroquine: Implications for Rheumatology. Nat. Rev. Rheumatol. 16, 155–166. 10.1038/s41584-020-0372-x 32034323

[B38] Tsai W.-P. Nara P. L. Kung H.-F. Oroszlan S. (1990). Inhibition of Human Immunodeficiency Virus Infectivity by Chloroquine. AIDS Res. Hum. Retroviruses 6, 481–489. 10.1089/aid.1990.6.481 1692728

[B39] Vilain A. Otis S. Forget A. Blais L. (2008). Agreement between Administrative Databases and Medical Charts for Pregnancy-Related Variables Among Asthmatic Women. Pharmacoepidem. Drug Safe. 17, 345–353. 10.1002/pds.1558 18271060

[B40] World Health Organization List of Essential Medications (2019). World Health Organization List of Essential Medications. Availableat: https://apps.who.int/iris/bitstream/handle/10665/273826/EML-20-eng.pdf?ua=1.

[B41] Zhao J.-P. Sheehy O. Gorgui J. Bérard A. (2017). Can We Rely on Pharmacy Claims Databases to Ascertain Maternal Use of Medications during Pregnancy?. Birth Defects Res. 109, 423–431. 10.1002/bdra.23604 28398706

[B42] Zhao J. P. Sheehy O. Bérard A. (2015). Regional Variations in the Prevalence of Major Congenital Malformations in Quebec: The Importance of Fetal Growth Environment. J. Popul. Ther. Clin. Pharmacol. 22, e198–210. 26567551

