# Supplementary material for: Chloroquine and Hydroxychloroquine Use During Pregnancy and the Risk of Adverse Pregnancy Outcomes Using Real-World Evidence
Source: Front Pharmacol. 2021 Aug 2;12:722511. doi: 10.3389/fphar.2021.722511 (PMC8366774; doi:10.3389/fphar.2021.722511)

**Supplementary File**

This supplementary file has been provided by the authors to give readers additional information about their work.

Supplement to: Chloroquine and hydroxychloroquine use during pregnancy and the risk of adverse pregnancy outcomes using real-world evidence: Anick Bérard, Odile Sheehy, Jin-Ping Zhao, Evelyne Vinet, Caroline Quach, Sasha Bernatsky.

**Content:**

**Table S1.** CQ/HCQ codes

**Table S2.** Other anti-malarial drug codes

**Table S3.** ICD-9 and ICD-10 diagnostic codes for major congenital malformation.

**Table S4.** List of diagnostic codes (ICD-9 and ICD-10) for malaria, lupus, and rheumatoid arthritis (RA) as well as diagnostic codes and medications used for the covariates including HIV drugs.

**Table S5.** CQ/HCQ prevalence, duration of use, and mean daily dosage (CQ equivalent).

**Table S6.** Association between CQ/HCQ exposure during the 1st trimester and organ specific defects (where possible).

**Table S7.** Specific major congenital malformations identified with CQ/HCQ use.

**Figure S1.** Quebec Pregnancy Cohort database linkage**.**

**Figure S2.** Quebec Pregnancy Cohort outcomes and babies.

**Table S1. CQ/HCQ codes**

| **Generic name** | **Brand name** | **Generic code** |
| --- | --- | --- |
| Chloroquine |  | 1833 |
| Hydroxychloroquine | *Plaquenil* | 4654 |

**Table S2.** Other anti-malarial drug codes

| **Generic name** | **Brand name** | **Generic code** |
| --- | --- | --- |
| Atovaquone |  | 47012 |
| Atovaquone/proguanil | *Malarone* | 47295 |
| Mefloquine | *Mefloquine* | 47062 |
| Primaquine | *Primaquine* | 43969 |
| Proguanil |  | 10452 |
| Sulfadoxine-pyriméthamine |  | 45522 |
| Halofantrine |  | 47252 |

**Table S3.** Major congenital malformation codes by organ systems.

| **Organ system** | **ICD-9 code** | **ICD-10 code** |
| --- | --- | --- |
| Nervous system | 740.0-742.9 | Q00.0-Q07.9 |
| Eye, ear, face and neck | 743.0-744.9  *Exclusions*: 743.6, 743.8, 744.1- 744.9* | Q10.0-Q18.9  *Exclusions: Q10.0-Q10.6, Q13.0, Q13.2, Q13.5, Q15.8, Q17.0-Q17.5, Q17.8, Q17.9, Q18.0-Q18.9* |
| Circulatory system | 745.0-747.9  *Exclusions: 747.0, 747.5* | Q20.0-Q28.9  *Exclusions: Q25.0, Q27.0* |
| Respiratory system | 748.0-748.9  *Exclusions: 748.2, 748.3* | Q30.0-Q34.9  *Exclusions: Q30.2, Q30.8, Q31-Q32, Q33.1* |
| Orofacial clefts | 749.0-749.2 | Q35.0-Q37.9  *Exclusion: Q35.7* |
| Digestive system | 750.0-750.9  *Exclusions: 750.0, 750.1, 750.2, 750.5, 750.6, 751.0, 751.5* | Q38.0-Q45.9  *Exclusions: Q38.1-Q38.6, Q40.0, Q40.1, Q43.0,*  *Q43.4-Q43.9* |
| Genital organs | 752.0-752.9  *Exclusions: 752.4, 752.5, 752.8* | Q50.0Q56.9  *Exclusions: Q52.2-Q52.8, Q53, Q54.4, Q55.1, Q55.2, Q55.6, Q55.8, Q55.9* |
| Urinary system | 753.0-753.9  *Exclusion: 753.6* | Q60.0-Q64.9  *Exclusions: Q61.0, Q62.7, Q63.3, Q64.2, Q64.3* |
| Musculoskeletal system | 754.0-756.9  *Exclusions: 754.0, 754.1, 754.7, 754.8, 756.0, 756.2* | Q65.0-Q79.9  *Exclusions: Q65.3-Q65.6, Q66.2, Q66.3, Q66.5-Q66.9, Q67.0-Q67.4, Q67.6-Q67.8, Q68.0. Q68.1, Q68.3-Q68.8, Q70.3, Q74.1,*  *Q75.0, Q75.2,*  *Q75.3, Q75.8,*  *Q76.0, Q76.5,*  *Q79.5, Q79.8* |
| Integument | 757.0-757.9  *Exclusions: 757.2-757.6, 757.8* | Q80.0-Q84.9  *Exclusions: Q81,*  *Q82.1- Q82.8,*  *Q83.2, Q83.3, Q83.8,*  *Q84.1- Q84.6, Q84.8* |
| Chromosomal | 758.0-758.8  *Exclusion:758.4* | Q90.0-Q99.2  *Exclusions: Q95.0, Q95.1* |
| Other | 758.9, 759.0-759.9  *Exclusion: 759.9* | Q85.0-Q89.9, Q99.8, Q99.9  *Exclusion: Q89.9* |

**Table S4.** List of diagnostic codes (ICD-9 and ICD-10) for malaria, lupus, RA, and diagnostic codes and medications used for the definitions of covariates including HIV drugs, alcohol and tobacco dependence.

| **Malaria** | **ICD-9 code** | **ICD-10 code** |
| --- | --- | --- |
| Plasmodium falciparum malaria  Paludism with malignant fever | 084.0 | B50 |
| Plasmodium vivax malaria  Paludism with malignant fever | 084.1 | B51 |
| Plasmodium malariae malaria  Paludism with fever | 084.2 | B52 |
| [Plasmodium ovale](https://fr.wikipedia.org/wiki/Plasmodium_ovale) malaria  Paludism with unknown fever | 084.3 |  |
| Other specified malaria | 084.4 | B53 |
| Mixed malaria | 084.5 |  |
| Unspecified malaria | 084.6 | B54 |
| Paludism induced | 084.7 |  |
| Paludism with complications du to Plasmodium falciparum (Blackwater fever) | 084.8 |  |
| Other paludism complications | 084.9 |  |
| **Lupus** |  |  |
| Systemic lupus erythematosus | 710.0 | M32 |
| Lupus erythematosus chronic | 695.4 | L93 |
| **Rheumatoid arthritis** |  |  |
| Rheumatoid arthritis and other inflammatory polyarthropathies | 714 | M05 |
| Rheumatoid arthritis | 714.0 |  |
| Felty's syndrome | 714.1 |  |
| Other rheumatoid arthritis with visceral or systemic involvement | 714.2 |  |
| Juvenile chronic polyarthritis | 714.3 | M08 |
| Chronic postrheumatic arthropathy | 714.4 |  |
| Other specified inflammatory polyarthropathies | 714.8 | M06 |
| Unspecified inflammatory polyarthropathy | 714.9 |  |

**Hypertension**

ICD-9 codes: 401.0-405.9, 642.0-642.9 and 796.2

ICD-10 codes: I10.0, I10.1, I15.0, I15.1, I15.2, I15.8, I15.9, O10, O11, O12, O13, O14, O15 and O16

Medication generic codes:

| **Generic name** | **Quebec generic code** |
| --- | --- |
| Clonidine | 10751 |
| Methyldopa | 6136 |
| Hydralazine | 4524 |
| Minoxidil | 41564 |
| Doxazosine | 45625 |
| Prazosin | 37742 |
| Terazosin | 45520 |
| Acebutolol | 45463 |
| Atenolol | 43670 – 46325 -46315 |
| Bisoprolol | 47355 |
| Carvedilol | 47199 - 46319 |
| Labetalol | 45243 |
| Metoprolol | 38275 – 46763 - 46780 |
| Nadolol | 40563 |
| Oxprenolol | 42162 |
| Pindolol | 39016 |
| Pindolol-HCTZ | 45408 |
| Propranolol | 8229 |
| Sotalol | 44866 |
| Timolol | 38314 |
| Amlodipine | 47006 |
| Amlodipine/Atorvastatine |  |
| Felodipine | 45624 |
| Nifedipine | 42708 – 46388 - 46469 |
| Nifedipine-AAS | 47751 |
| Nimodipine | 45532 |
| Diltiazem | 43228 - 47247 |
| Verapamil | 40550 - 46573 |
| Verapamil-Trandolapril | 47440 |
| Benazepril | 47049 |
| Captopril | 42071 |
| Cilazapril | 47056 |
| Cilazapril-HCTZ | 47320 |
| Enalapril | 45476 |
| Enalapril-HCTZ | 45572 |
| Fosinopril | 47002 |
| Lisinopril | 45576 |
| Lisinopril-HCTZ | 47040 |
| Perindopril | 47117 - 46258 |
| Perindopril-Indapamide | 47449 |
| Quinapril | 45629 |
| Quinapril-HCTZ | 47301 |
| Ramipril | 47079 - 46216 |
| Ramipril-HCTZ | 47655 |
| Trandolapril | 47250 |
| Trandolapril/Verapamil |  |
| Candesartan | 46529 - 47309 |
| Candesartan-HCTZ | 46760 - 47412 |
| Eprosartan | 47389 |
| Eprosartan-HCTZ | 47534 - 47532 |
| Irbesartan | 46459 - 47282 |
| Irbesartan-HCTZ | 47354 |
| Losartan | 47135 – 46284 - 46441 |
| Losartan-HCTZ | 47207 |
| Olmesartan medoxomil | 47763 |
| Olmesartan medoxomil-HCTZ | 47764 |
| Telmisartan | 47333 - 46587 |
| Telmisartan-HCTZ | 47413 |
| Telmisartan/Amlodipine | N.A. |
| Valsartan | 46418 - 47259 |
| Valsartan-HCTZ | 47369 |
| Spironolactone | 9100 - 46572 |
| Ethacrynique | 3562 |
| Furosemide | 4173 |
| Amiloride | 41759 |
| Amiloride-HCTZ | 41772 |
| Hydrochlorothiazide | 4537 |
| Chlorthalidone | 1976 |
| Indapamide | 43397 |
| Metolazone | 19440 |
| Amiloride-HCTZ | 41772 |
| Spironolactone-HCTZ | 38158 |
| Triamtérène-HCTZ | 38197 |
| Triamtérène | 9763 |
| Amlodipine-Atorvastatine | 47609 |
| Aliskirene | 47706 |
| Aliskirene-HCTZ | 47823 |
| Excluding the medication on the following formulation: | |
| Formulation | Code |
| Ophthalmic powder | 1479 |
| Ophthalmic ointment | 1624 |
| Ophthalmic solution | 2204 |
| Ophthalmic and Optic Solution | 2233 |
| Ophthalmic suspension | 2784 |
| Ophthalmic and Optic Suspension | 2813 |
| Ophthalmic Irrigation Solution | 3480 |
| Ophthalmic gel | 5365 |
| Ophthalmic Gel Solution | 5599 |

**Diabetes**

ICD-9 codes: 250.0-250.9, 271.4 and 790.2

ICD-10 codes: E10-E14 and R73.0

Medication generic codes:

| **Generic name** | **Quebec generic code** |
| --- | --- |
| Metformin | 5824 - 47208 |
| Glucagon | 4238 |
| Chlorpropamide | 1937 |
| Glyburide | 4264 |
| Tolbutamide | 9672 - 15184 |
| Gliclazide | 46056 - 47329 |
| Glimepiride | 46799 - 47427 |
| Acarbose | 46300 - 47151 |
| Pioglitazone | 46678 - 47392 |
| Rosiglitazone | 47371 - 46642 |
| Rosiglitazone/Metformin | 46862 |
| Rosiglitazone/Glimepiride | 47652 |
| Nateglinide | 46810 |
| Repaglinide | 47357 - 46568 |
| Saxagliptine | 47817 |
| Sitagliptine | 47715 |
| Sitagliptine/Metformin | 47807 - 47832 |
| Insulin aspart | 46798 - 47424 |
| Insulin aspart/ Insulin aspart protamin |  |
| Insulin glulisine | 47749 |
| Insulin isophane bio-synthetic | 44164 |
| Insulin lispro | 46322 - 47206 |
| Inslulin zinc cristallin bio-synthetic | 44489 |
| Insulins zinc cristallin et isophan bio-synthetic | 45531 |
| Insulin aspart/insulin aspart protamin | 47615 |
| Insulin détémir | 47586 |
| Insulin glargine | 47536 |
| Insulin lispro/insulin lispro protamin | 47426 |
| Insulin globin zinc | 4823 |
| Insulin sulfate | 4888 |
| Insulin zinc cristallin (porc) | 18296 |
| Insulin protamine zinc (beef) | 18309 |
| Insulin protamin zinc (porc) | 18322 |
| Insulin isophan (porc) | 18335 |
| Insulin isophan (beef) | 18348 |
| Insulin (beef et porc) | 39120 |
| Insulin isophan (beef et porc) | 39133 |
| Insulin protamin zinc (beef et porc) | 39146 |
| Insulin semilente (beef et porc) | 39159 |
| Insulin (beef et porc) | 39172 |
| Insulin zinc cristallin (beef et porc) | 39185 |
| Insulin isophan (beef) * | 39458 |
| Insulin protamin zinc (beef) * | 39484 |
| Insulin protamin zinc (porc) * | 39497 |
| Insulin zinc cristallin (beef) * | 39523 |
| Insulin (porc) | 41655 |
| Insulin zinc cristallin (porc)/ insulin isophane (porc) | 43033 |
| Insulin zinc cristallin (beef) | 43735 |
| Insulin isophan semi-synthetic human sequence | 44151 |
| Insulin semi-synthetic human sequence | 44476 |
| Insulin zinc cristallin semi-synthetic human sequence | 44502 |
| Insulin semi-synthetic human sequence | 44996 |
| Insulins isophan et zinc cristallin semi- synthetic human sequence | 45405 |
| Insulin bio- synthetic human sequence | 45415 |
| Insulin bio- synthetic human sequence | 45483 |
| Insulins isophan et zinc cristallin bio- synthetic human sequence | 45511 |
| Insulins zinc cristallin et isophan semi- synthetic human sequence | 45534 |
| Insulin zinc cristallin (beef et porc) | 46536 |
| Insulin isophan (beef et porc) | 46537 |
| Insulin (beef et porc) | 46538 |
| Insulin isophan (human)/ insulin injectable(human) | 46592 |
| Insulin isophan (human) | 46602 |
| Insulin injectabl (human) | 46603 |
| Insulin lispro/insulin isophan (human) | 46607 |
| Insulin zinc cristallin (porc) * | 47004 |
| Insulin lispro/ insulin lispro protamin | 47426 |
| Alogliptine |  |
| Alogliptine/Metformin |  |
| Canagliflozine |  |
| Dipagliflozine |  |
| Glidazide |  |
| Glimepiride |  |
| Linagliptine |  |
| Linagliptine/Metformin |  |
| Liragludine |  |

**Asthma**

ICD-9 codes: 493.0, 493.1, 493.3, 493.4, 493.5, 493.6, 493.7, 493.8 and 493.9

ICD-10 codes: J45.0, J45.8, J45.1 and J45.9

Medication generic codes

| **Generic name** | **Quebec generic code** |
| --- | --- |
| Aminophylline | 364, 46428 |
| Beclomethasone | 780* |
| Budesonide | 45499* |
| Budesonide/ formoterol | 47428, 46800 |
| Cromoglicate sodique | 39419, 47315 |
| Cromoglycate disodique | 2223 |
| Epinephrine | 3380 |
| Epinephrine | 3406 |
| Epinephrine racemic | 3419 |
| Fenoterol | 38548 |
| Flunisolide | 38730* |
| Fluticasone | 47050*,46435* |
| Formoterol | 47231* |
| Formoterol | 47271,46430 |
| Formoterol / budesonide | 47428 |
| Ipratropium (bromure d') | 43124, 46640 |
| Ipratropium (bromure d')/ salbutamol (sulfate de) | 47186, 46302 |
| Isoproterenol (chlorhydrate d') | 5083 |
| Isoproterenol (chlorhydrate d')/ phenylephrine (bitartrate de) | 5096 |
| Isoproterenol (chlorhydrate d')/ phenylephrine (chlorhydrate de) | 5109 |
| Isoproterenol (sulfate d') | 5070 |
| Ketotifene (fumarate de) | 45555, 46752 |
| Montelukast sodique | 47303, 47302, 46467 |
| Nedocromil sodique | 47033, 45563, 46463 |
| Orciprenaline (sulfate d') | 6721 |
| Oxtriphylline | 43475 |
| Pirbuterol (acetate de) | 47153,46299 |
| Procaterol hemihydrate (chlorhydrate de) | 45547 |
| Salbutamol | 10530 |
| Salbutamol (sulfate de) | 33634,46737 |
| Salmeterol (xinafoate de ) / fluticasone (propionate de ) | 47335,46597 |
| Salmeterol (xinafoate de) | 47112,46247 |
| Terbutaline (sulfate de) | 34180 |
| Theophylline | 9464,46847 |
| Theophylline (aminoacetate calcique de) | 9490 |
| Theophylline (aminoacetate sodique de) | 9503 |
| Theophylline/dextrose | 44944 |
| Triamcinolone (acetonide) | 9737* |
| Zafirlukast | 47266,46401 |
| Ciclesonide | 47626* |
| Momethasone | 45581 |
| Momethasone/Formeterol |  |
| Zolair |  |

For code with a * use only the following formulations:

| **Formulation** | **Code** |
| --- | --- |
| Powder aerosol | 1305 |
| Powder aerosol with applicator | 1334 |
| Aerosol solution | 1856 |
| Aerosol solution with applicator | 1885 |
| Solution for Inhalation | 1972 |
| Suspension aerosol | 2610 |
| Suspension aerosol with applicator | 2639 |
| Inhalation powder with applicator | 5563 |
| Inhalation powder | 5564 |
| Oral spray | 5584 |
| Gel | 5619 |
| Powder for solution for inhalation | 5634 |

**Thyroid disorders**

ICD-9 codes : 244.0-244.9 and 242.9

ICD-10 codes : E01, E02, E03 and E05

Medication generic codes:

| **Generic name** | **Quebec generic code** |
| --- | --- |
| Levothyroxine sodique | 5252 - 46574 |
| Liothyronine sodique/ levothyroxine sodique | 33842 |
| Liothyronine sodique | 5317 – 46457 - 46474 |
| Methimazole | 40836 |
| Propylthiouracile | 8242 |

**Tobacco dependence**

ICD-9 codes: 305.0, 305.1 and 649.0

ICD-10 codes: F17, O99.33, Z71.6, and Z72.0

**Alcohol dependence**

ICD-9 codes: 303, 305.0 and 980

ICD-10 codes: F10, O99.31

**Other drug dependences**

ICD-9 codes: 304.0, 304.2, 304.3, 304.4, 304.5, 304.6, 304.7, 304.8 and 304.9

ICD-10 codes: F11, F12, F14, F15, F16, F18, F19

HIV drugs authorized in Canada

| **Generic name** | **Brand name** | **Dose and dosage** | **Generic code** |
| --- | --- | --- | --- |
| **Combined medication with specific dosage** | | | |
| Éfavirenz / emtricitabine / tenofovir disoproxil (fumarate) | ***Atripla*** | 600mg/200mg/300mg  1 co die | 47724 |
| Bictegravir + tenofovir alafenamide + emtricitabine | ***Biktarvy*** | 50mg/25mg/200mg  1 co die | N.A. |
| Tenofovir disoproxil fumarate / emtricitabine/ rilpivirine | ***Complera***  ***Odefsey*** | 300mg/200mg/25mg  1 co die  25mg/25mg/200mg  1 co die | 47895 |
| Doravirine /lamivudine/tenofovir disoproxil fumarate | ***Delstrigo*** | 100mg/300mg/300mg  1 co die | N.A. |
| Elvitegravir/cobicistat /tenofovir alafenamide /emtricitabine | ***Genvoya*** | 150mg/150mg/10mg  1 co die | 47946 |
| Dolutegravir/rilpivirine | ***Juluca*** | 50mg/25mg  1 co die | N.A. |
| Elvitegravir/cobicistat / tenofovir disoproxil fumarate/ emtricitabine | ***Stribild*** | 150mg/150mg/300mg  /200 mg  1 co die | 47946 |
| Darunavir/cobicistat/ tenofovir alafenamide/ emtricitabine | ***Symtuza*** | 800mg/150mg/10mg/ 200 mg  1 co die | N.A. |
| Dolutegravir/abacavir/lamivudine | ***Triumeq*** | 50mg/600mg/300mg  1 co die | 48043 |
| **Other combinations** | | | |
| Lamivudine/zidovudine (AZT) | ***Combivir*** | 150mg/300mg  1 co bid | 46520  47310 |
| Tenofovir alafenamide/ emtricitabine | ***Descovy*** | 10mg/200mg or  25mg/200 mg  1 co die | 47605 |
| Abacavir/lamivudine | ***Kivexa*** | 600mg/300mg  1 co die | 47576 |
| Darunavir/cobicistat | ***Prezcobix*** | 800mg/150mg  1 co die | 48026 |
| Lamivudine + AZT + abacavir | ***Trizivir*** | 300mg/150mg/300mg  1 co bid | 46769  47422 |
| Emtricitabine/tenofovir disoproxil fumarate | ***Truvada*** | 200mg/300mg | 47605 |
| **Integrase inhibitors** |  |  |  |
| Raltegravir | ***Isentress***  ***Isentress HD*** | 400mg  1 co bid  600mg  2 co die | 47700 |
| Dolutegravir | ***Tivicay*** | 50mg  1 co die or bid | 47984 |
| Elvitegravir | ***Vitekta*** |  |  |
| **Protease inhibitors** | | | |
| Tipranavir | ***Aptivus*** | 250mg  2 co. bid | 47585 |
| Lopinavir/ ritonavir | ***Kaletra*** | 200mg/50mg  2 co. bid or  4 co. die | 46714  47397 |
| Ritonavir | ***Norvir*** | 100mg  6 co. bid | 46313  47170 |
| Darunavir | ***Prezista*** | 75mg  200mg  800mg 1 co bid  600mg exception | 47623  47632 |
| Atazanavir | ***Reyataz*** | 150mg  200mg - 2 co die  300mg - 1 co die | 47479 |
| Saquinavir | ***Invirase*** | 200mg  500mg | 46519  47158  47306 |
| Nelfinavir | ***Viracept*** | 250mg  625mg | 46475  47293 |
| Fosamprenavir | ***Telzir*** | 700mg  50mg/ml | 47543 |
| Indinavir | ***Crixivan*** |  | 46314  47167 |
| **CCR5 antagonist** |  |  |  |
| Maraviroc | ***Celsentri*** | 150mg  1 co bid  300mg  1 co ou 2 co bid | 47701 |
| **Fusion inhibitor** | | | |
| Enfuvirtide pour injection | ***Fuzeon*** | 108mg  Bid | 47466 |
| **Nucleoside/nucleotide reverse transcriptase inhibitors (NRTIs)** | | |  |
| Lamivudine | ***3TC*** | 100mg  3 co. bid ou tid  150mg  1 co bid  300mg  1 co die  10mg/ml | 46514  47147 |
| Retrovir | ***AZT*** | 100mg  3 co bid or 2 co tid  80mg/ml | 45589 |
| Tenofovir disoproxil fumarate | ***Viread*** | 300mg  1 co die | 47512 |
| Abacavir | ***Ziagen*** | 300mg  1 co bid or 2 co die  20mg/ml | 46564  47324 |
| Stavudine | ***Zerit*** | 15mg  20mg  30mg  40mg | 46311  47171 |
| Didanosine | ***Videx EC*** | 125mg  200mg  250mg  400mg | 45611  46581 |
| Adefovir dipivoxil | ***Hepsera*** | 10mg | 47607 |
| Emtricitabine | ***Emtriva*** |  | N.A. |
| Tenofovir alafenamide | ***Vemlidy*** |  | N.A. |
| Zidovudine | ***Retrovir*** |  | 45589 |
| **Non-nucleoside reverse transcriptase inhibitors (NNRTIs)** | | | |
| Rilpirivine | ***Edurant*** | 25mg  1 co die | 47876  47891 |
| Etravirine | ***Intelence*** |  | 47727 |
| Doravirine | ***Pifeltro*** | 100mg  1 co die | N.A. |
| Efavirenz | ***Sustiva*** | 50mg  200mg  3 co. die  600mg  1 co. die | 47323 |
| Nevirapine | ***Viramune***  ***Viramune XR*** | 200mg  1 co bid  400mg  1 co die | 46505  47294 |
| Delavirdine | ***Rescriptor*** | 100mg | 47313 |
| Rilpivirine | ***Edurant*** | 25mg | 47876  47891 |

**Table S5.** CQ/HCQ prevalence, duration of use, and mean daily dosage (CQ equivalent).

| **CQ/HCQ exposure prevalence** | **Pregnancies**  **(n)** | **Percentage (%)** |
| --- | --- | --- |
| During pregnancy (n = 288)  Chloroquine  Hydroxychloroquine | 183  105 | 63.54  36.46 |
| During the 1st trimester (n = 218)  Chloroquine  Hydroxychloroquine | 126  92 | 57.80  42.20 |
| During the 2nd/3rd trimester (n = 159)  Chloroquine  Hydroxychloroquine | 87  72 | 54.72  45.28 |

|  | **Pregnancies** | **Number of days exposed** | **Median**  **(min - max)** |
| --- | --- | --- | --- |
| **CQ/HCQ exposure duration** |  | **mean ± SD** |  |
| During pregnancy | 288 | 71.82 ± 70.47 | 42 (1 - 294) |
| During the 1st trimester | 218 | 41.91 ± 27.56 | 33.5 (1 - 98) |
| During the 2nd/3rd trimesters | 159 | 71.82 ± 52.07 | 49 (1 - 196) |

| **CQ equivalent dosage** | **Pregnancies (n)** | **Mean daily dose (mg) ± SD** | **Mean number of prescriptions filled** |
| --- | --- | --- | --- |
| Any time during pregnancy | 288 | 204.26 ± 155.64 | 2.52 ± 3.27 |
| Exposed during the first trimester | 218 | 218.45 ± 154.60 | 1.74 ± 4.29 |
| Exposed during the 2nd/3rd trimesters | 159 | 215.62 ± 157.71 | 2.69 ± 2.54 |

**Table S6.** Association between CQ/HCQ exposure during the 1st trimester and organ specific defects (where possible).

|  | **Nervous system malformations** | | | |
| --- | --- | --- | --- | --- |
| **Variables** | **Yes**  **n=2,237** | **No**  **n=231,511** | **Crude OR**  **(95% CI)** | **Adjusted* OR**  **(95% CI)** |
|  | **n (%)** | |  |  |
| CQ/HCQ  Other antimalarial medications | 4 (0.18)  1 (0.04) | 214 (0.09)  128 (0.06) | 1.95 (0.72-5.25)  0.83 (0.12-5.61) | 1.87 (0.68-5.17)  0.81 (0.12-5.51) |
|  | **Circulatory system malformations including heart defects** | | | |
|  | **Yes**  **n=7,010** | **No**  **n=226,738** | **Crude OR**  **(95% CI)** | **Adjusted* OR**  **(95% CI)** |
| CQ/HCQ  Other antimalarial medications | 8 (0.11)  5 (0.07) | 210 (0.09)  124 (0.05) | 1.19 (0.58-2.44)  1.29 (0.53-3.12) | 0.83 (0.38-1.79)  1.25 (0.52-3.01) |
|  | **Genital system malformations** | |  |  |
|  | **Yes**  **n=3,499** | **No**  **n=230,249** | **Crude OR**  **(95% CI)** | **Adjusted* OR**  **(95% CI)** |
| CQ/HCQ  Other antimalarial medications | 1 (0.03)  4 (0.11) | 217 (0.09)  125 (0.05) | 0.30 (0.04-2.13)  2.15 (0.80-5.78) | 0.36 (0.05-2.66)  2.05 (0.76-5.55) |
|  | **Musculoskeletal system malformations** | | | |
|  | **Yes**  **n=14,351** | **No**  **n=219,397** | **Crude OR**  **(95% CI)** | **Adjusted* OR**  **(95% CI)** |
| CQ/HCQ  Other antimalarial medications | 15 (0.10)  5 (0.03) | 203 (0.09)  124 (0.06) | 1.14 (0.68-1.91)  0.63 (0.26-1.50) | 0.99 (0.58-1.69)  0.62 (0.26-1.48) |

*Adjusted for all variables in **Tables 2, 3 or 4**.

**Table S7.** Specific major congenital malformations identified with CQ/HCQ use.

| 4 Nervous system defects | 1 microcephaly  1 congenital reduction deformities of brain  2 other specified congenital malformations of brain |
| --- | --- |
| 8 Circulatory system defects | 1 ventricular septal defect with stenosis of pulmonary artery defect  1 ventricular septal defect alone  2 atrial septal defect  1 atrial septal defect with other congenital malformations of cardiac chambers and connections  1 atrial septal defect with atrioventricular septal defect and congenital mitral stenosis  1 atrioventricular septal defect with hypoplastic left heart syndrome  1 congenital malformation of aortic and mitral valves unspecified |
| 1 Genital defect | 1 congenital absence of ovary |
| 15 Musculoskeletal defects | 1 congenital metatarsus (primus) varus  1 congenital metatarsus (primus) varus with craniosynostosis  1 congenital deformity of knee  1 congenital hypertrophic pyloric stenosis with macrocephaly  1 congenital musculoskeletal deformities of skull, face, and jaw unspecified with other congenital malformations of lower limb(s) including pelvic girdle  1 congenital malformations of lower limb(s) unspecified  1 congenital deformity of sternocleidomastoid muscle with unspecified congenital malformation of limb(s)  5 congenital anomalies of skull and face bones alone  1 other congenital deformities of feet  2 other specified congenital musculoskeletal deformities |

**Figure S1.** Quebec Pregnancy Cohort database linkage**.**

**Figure S2.** Quebec Pregnancy Cohort outcomes and babies.


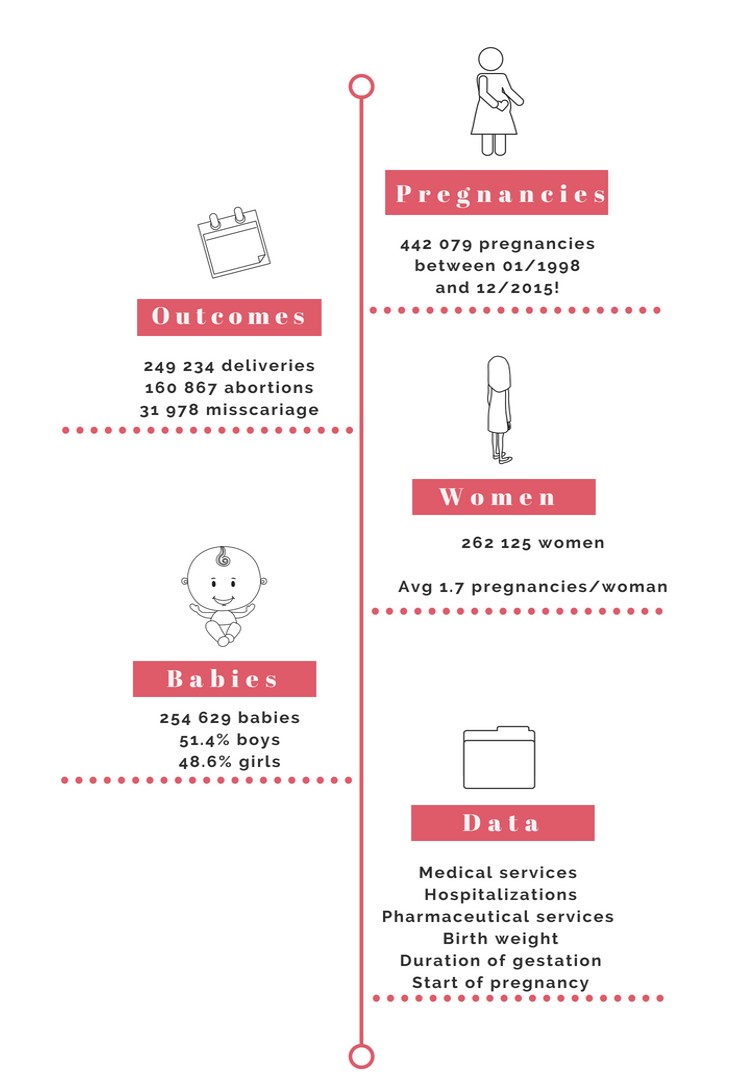

Supplement: Supplementary file 1 [file DataSheet1.doc]
